# Maintenance of spontaneous breathing at an intensity of 60%–80% may effectively prevent mechanical ventilation-induced diaphragmatic dysfunction

**DOI:** 10.1371/journal.pone.0229944

**Published:** 2020-03-04

**Authors:** Zujin Luo, Silu Han, Wei Sun, Yan Wang, Sijie Liu, Liu Yang, Baosen Pang, Jiawei Jin, Hong Chen, Zhixin Cao, Yingmin Ma

**Affiliations:** 1 Department of Respiratory and Critical Care Medicine, Beijing Engineering Research Center of Respiratory and Critical Care Medicine, Beijing Institute of Respiratory Medicine, Beijing Chao-Yang Hospital, Capital Medical University, Beijing, China; 2 Department of Pathology, Beijing Chao-Yang Hospital, Capital Medical University, Beijing, China; University of Tennessee Health Science Center College of Graduate Health Sciences, UNITED STATES

## Abstract

Controlled mechanical ventilation (CMV) can cause diaphragmatic motionlessness to induce diaphragmatic dysfunction. Partial maintenance of spontaneous breathing (SB) can reduce ventilation-induced diaphragmatic dysfunction (VIDD). However, to what extent SB is maintained in CMV can attenuate or even prevent VIDD has been rarely reported. The current study aimed to investigate the relationship between SB intensity and VIDD and to identify what intensity of SB maintained in CMV can effectively avoid VIDD. Adult rats were randomly divided according to different SB intensities: SB (0% pressure controlled ventilation (PCV)), high-intensity SB (20% PCV), medium-intensity SB (40% PCV), medium-low intensity SB (60% PCV), low-intensity SB (80% PCV), and PCV (100% PCV). The animals underwent 24-h controlled mechanical ventilation (CMV). The transdiaphragmatic pressure (Pdi), the maximal Pdi (Pdi max) when phrenic nerves were stimulated, Pdi/Pdi max, and the diaphragmatic tonus under different frequencies of electric stimulations were determined. Calpain and caspase-3 were detected using ELISA and the cross-section areas (CSAs) of different types of muscle fibers were measured. The Pdi showed a significant decrease from 20% PCV and the Pdi max showed a significant decrease from 40% PCV (P<0.05). In vivo and vitro diaphragmatic tonus exhibited a significant decrease from 40% PCV and 20% PCV, respectively (P<0.05). From 20% PCV, the CSAs of types I, IIa, and IIb/x muscle fibers showed significant differences, which reached the lowest levels at 100% PCV. SB intensity is negatively associated with the development of VIDD. Maintenance of SB at an intensity of 60%-80% may effectively prevent the occurrence of VIDD.

## Introduction

Invasive mechanical ventilation is an important life-saving intervention approach in the management of respiratory failure in the intensive care unit (ICU). However, prolonged mechanical ventilation is likely to cause diaphragmatic atrophy and contractile dysfunction, which is collectively referred to ventilator-induced diaphragmatic dysfunction (VIDD). Currently, the widely-accepted explanation for the mechanism underlying VIDD is that mechanical ventilation provides excessive pressure support, which decreases or even inhibits the respiratory drive of the patient, thereby causing disuse atrophy of the diaphragm within a short time period [1, 2]. VIDD is a major contributor to ventilator weaning failure [3, 4]. Prolongation of invasive mechanical ventilation increases the risk of re-admission to ICU [5] and one-year mortality [6].

The first prospective study on the impact of controlled mechnical ventilation (CMV) on diaphragmatic function in rats was reported by Le Bourdelles et al in 1994, according to which 48-h CMV led to atrophy and weakness of the diaphragm [7]. Since then, numerous animal studies have proved that inappropriate CMV can induce VIDD [8–13]. Human studies have also revealed atrophy of diaphragmatic fibers caused by complete CMV [14]. In seven patients receiving complete CMV, the thickness of the diaphragm decreased at a rate of 6% per day [15]. Along with the presence of VIDD, CMV-caused variations in the levels of proteolysis-related markers, such as Calpains and Caspase-3, can occur; these proteases promote the release of actomyosin from sarcomere [16–18]. The cleaved actomyosin is then degraded via the ubiquitin-proteasome system, ultimately leading to diaphragmatic proteolysis. The inhibition of the activity of Caspase-3 and Calpains can mitigate diaphragmatic contractile dysfunction and atrophy [19, 20]. During controlled ventilation, diaphragm nerve stimulation improves diaphragm contraction, which noticeably improves mitochondrial function, reduces oxidative stress reaction and strengthens diaphragmatic tonus [21–23]. Intermittent SB during CMV can improve the decreased IIx/b fiber cross-section area, weakened diaphragmatic contractility, and diaphragmatic protein levels [24]. However, even in the case where spontaneous breathing (SB) is allowed, if the support from pressure support ventilation (PSV) is too high, the oxidative stress reaction of the diaphragm may also be enhanced, which increases the activity of 20S proteasome, Calpain and Casepase-3; consequently, the cross-section area of diaphragmatic fibers is reduced and the muscle strength is noticeably weakened [13, 25]. Here, a controversy arises: Even SB is maintained, its effect on VIDD varies. Presumably, the controversy is primarily caused by the use of different respiratory support and SB intensities. Then, what intensity of SB can effectively prevent VIDD and what relationship between SB intensity and VIDD have become the keys to solve this controversy.

In this study, we aimed to investigate at what intensity of SB VIDD can be effectively prevented and to explore the association between SB intensity and VIDD. To achieve these goals, CMV animal models of different SB intensities were established, and then diaphragmatic atrophy related indices were compared.

## Materials and methods

### Model establishment

Healthy adult Sprague-Dawley rats (9 weeks) were purchased from Beijing Vital River Laboratory Animal Technology Co., Ltd. (Beijing, China), They were randomly divided into six groups (n = 10; equal sex) according to SB intensity: complete SB (100% pressure controlled ventilation (PCV)), high-intensity SB (20% PCV), medium-intensity SB (40% PCV), medium-low intensity SB (60% PCV), low-intensity SB (80% PCV), and complete PCV (100% PCV).

The animals were anesthetized with an intraperitoneal (IP) injection of 1% sodium pentobarbital (90 mg/kg). Then, they were subjected to tracheotomy and CMV. Bilateral jugular veins were cannulated for continuous infusion of physiological saline (noradrenaline was administered to maintain hemodynamic stability when necessary). The carotid artery was opened and the arterial blood pressure was monitored. After operation, the animal was subjected to body plethysmography followed by PCV. The SB frequency of the normal rats was approximately 100 times/min. By adjusting the pumped dosage of pentobarbital sodium dynamically, the SB frequency was decreased from 100 times/min to 80, 60, 40, 20, 0 times/min, respectively, and six rat models of different SB intensities were established. The animals were subjected to invasive positive pressure ventilation (RWD407; RWD Life Science, Shenzhen, China), and the parameters were set as follows: positive end expiratory pressure (PEEP), 1 cmH_2_O; pressure control (PC), 8 cmH_2_O above the PEEP; controlled ventilatory frequency, 0 times/min (0% PCV), 20 times/min (20% PCV), 40 times/min (40% PCV), 60 times/min (60% PCV), 80 times/min (80% PCV), and 100 times/min (100% PCV) for SB frequency at 100, 80, 60, 40, 20, and 0 times/min, respectively. Under the required pressure, the rats were ventilated continuously for 24 h.

During ventilation, routine intensive care measures, such as body turning, body temperature maintenance, inspired air warming and humidification and nutrition supply maintenance, were performed for the animals. Throughout the study, data measurement and detection were performed by two independent experienced researchers, and they were blinded to animal group assignment.

The procedures of this study gained approval from the Institute of Animal Ethics of Capital Medical University (No. CMU-2017-2-25).

### Measurement of Pdi and maximal transdiaphragmatic pressure (Pdi max)

Pdi refers to the difference between the intrapleural pressure (Ppl) and the intra-abdominal pressure (Pab). After 24-h ventilation, esophageal intubation was performed, and then a pressure sensor was connected for Pdi measurement. A 1 cm-long transverse incision was made to expose the xihoid bone and the ventral surface of the diaphragm. A thin-walled rubber balloon used for Pab measurement was placed beneath the convex part of the diaphragm and the pressure sensor was connected. Pdi was obtained based on the measured Ppl and Pab. Then, bilateral cervical phrenic nerves were isolated and then stimulated electrically at a frequency of 100 Hz with a wave width of 0.1 ms and an intensity of 40 V for 5 s. The airway was closed. The Ppl and Pab were re-obtained for determination of Pdi max in vivo (an index to reflect the maximal contractility of the diaphragm).

### Diaphragmatic tonus detection

#### In vivo diaphragmatic tonus

**Fig 1:**
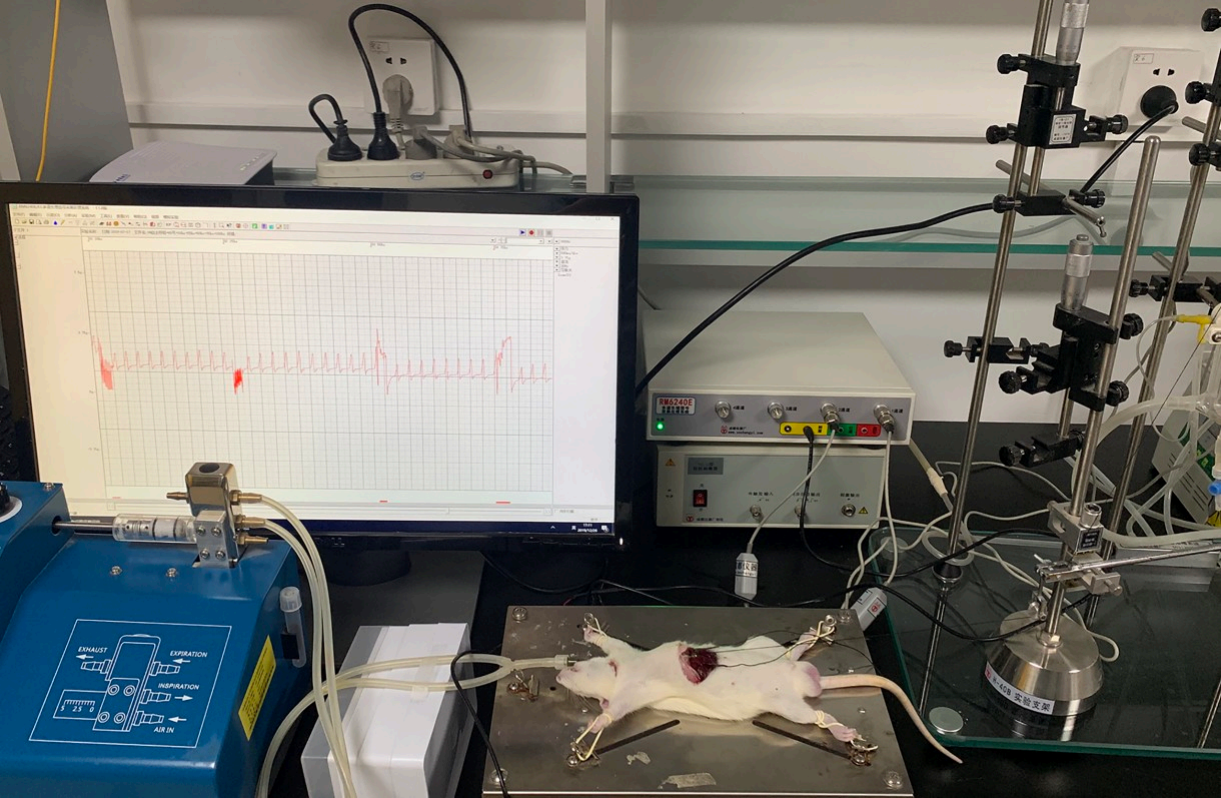
Equipment for in vivo diaphragmatic tonus measurement.

In vivo diaphragmatic tonus was measured as shown in Fig 1. After Pdi measurement, a steel hook and a stimulating electrode were attached near the central tendon of the diaphragm. Their exposed ends were connected to a tension sensor and a stimulator, respectively. Then, the devices were connected to the biological function test system. Electric stimulations at 10, 25, 50, 75, and 100 Hz were subsequently performed. The stimulation voltage was 1.5 times the threshold voltage (5 V). The wave width was 10 ms, and the stimulation time limit was 500 ms with the time interval between stimulations of 1 min. The diaphragmatic contractility under each stimulation frequency was measured.

#### In vitro diaphragmatic tonus

The animals were sacrificed via bloodletting under anesthesia. The diaphragm was exposed immediately. A 0.4 cm×1.5 cm muscle strip was taken from the left hemi-diaphragm (the central tendon and rib were retained). The strip was suspended in the Magnus bath which contained Kreb solution at 27°C and was ventilated with 95% CO_2_ and 5% O_2_. The rib part of the strip was fixed onto the ventilation hook with a frog heart clip, and the central tendon part was ligated with suture and connected to a tonotransducer (JZJ01; Chengdu Instrument Factory, Chengdu, China). A platinum stimulating electrode was inserted directly into muscle fibers. The tonotransducer was fixed onto the fine adjustment knob for the convenience of initial length adjustment for the strip. After a proper initial length (Lo) was reached, the strip was let stand for 20 min, and then the mechanical indices were measured. For each time of stimulation, the wave width was 2 ms and the voltage was 90 V. The time interval between strings of stimuli was 30 s at least. Each string of stimuli was 400 ms in length, and electric stimulation was performed at a frequency of 10, 20, 40, 60, and 100 Hz, respectively. Based on the obtained diaphragmatic contractility, a tension-frequency curve was plotted. The values were standardized according to the cross-section area (CSA) of the strip. After each strip tension experiment, the diaphragm was weighed. The CSA was calculated based on the following formula:

CSA (cm^2^) = muscle strip weight (g)/the length of the strip (era)×muscle density of the diaphragm (1.056 g/cm^3^, according to the literatures [25, 26])

### Pathological observation and detection of the activity of hydrolytic metabolic enzymes

After the animals were sacrificed, the diaphragm was immediately taken. The left hemi-diaphragm was used for pathological observation, whereas the right counterpart was used for detection of the activity of calpain and caspase-3 [26–28].

#### Light microscopy

The left diaphragm was immediately placed in glutaric acid fixation fluid and pathological sections were made. The variations in diaphragmatic fibers were observed using light microscopy (20×; Olympus BX53, Japan) [29, 30].

#### Enzyme-linked immunosorbent assays (ELISA)

The activity of calpain and caspase-3 was determined using ELISA. Briefly, 100 μl of standard preparation and of samples was added into the pores. The plate was blocked for 1-h incubation at 37°C. The liquid was removed. Approximately 100 μl of primary antibodies was applied into each pore for 1-h incubation at 37°C. The liquid in the pores was removed and the samples were washed thrice. Approximately 100 μl of enzyme-conjugated antibody was added into each pore for 30-min incubation at 37°C. The liquid was removed and the samples were washed five times. Then, 3,3',5,5'-tetramethylbenzidine (TMB) substrate solution at 90 μl was added for 10-min incubation at 37°C. Finally, termination solution at 50 μl was added to terminate reactions. Colorimetry was performed with an automatic microplate reader (iMARK; Bio-Rad, Hercules, CA, USA) at a wavelength of 450 mn. A standard curve was plotted according to the absorbance of the standard product as well as its recorded concentrations. The concentrations of the target proteases were calculated based on the standard curve.

### Statistical analysis

Data were processed by SPSS 25.0 and measurement data that satisfied normal distribution were presented as mean ± SD. One-factor analysis of variance was performed to compare among groups followed by a post-hoc least significant difference (LSD) test. P<0.05 was considered statistically significant.

## Results

### Rat models

In each group, 10 adult rats were included. The groups did not show significant differences in weight and the overall ventilation frequency (P>0.05). There were significant differences in the SB frequency among the groups (P<0.05; Table 1).

**Table 1:** Baseline data of the animals.

Variable	0% PCV	20% PCV	40% PCV	60% PCV	80% PCV	100% PCV	P value
Body weight (g)	299±32	306±29	354±77	319±40	303±40	313±27	0.093
Spontaneous respiratory rate/min	101±1	80±1	61±1	41±2	21±1	0±0	<0.0001
Total ventilation frequency/min	101±1	98±6	101±1	101±1	101±1	100±1	0.259

### Variations in Pdi and muscle tonus

**Fig 2:**
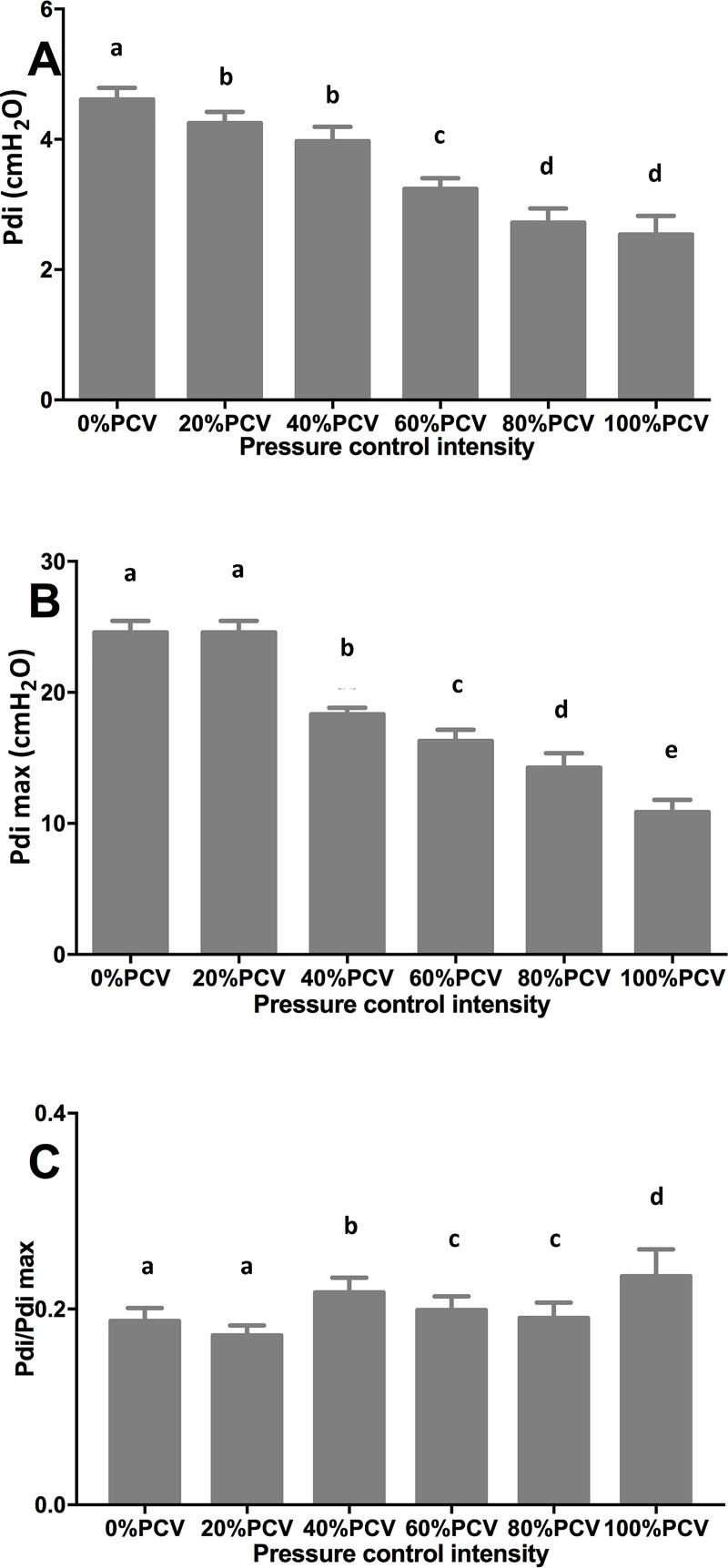
Transdiaphragmatic pressure (Pdi; A), the maximal transdiaphragmatic pressure (Pdi max; B) and Pdi/Pdi max (C) after 24-h controlled mechanical ventilation at different intensities. A different letter indicates a significant difference (P<0.05).

With the increase in the PCV intensity, the Pdi gradually decreased (Fig 2A). Compared with the 0% PCV group, other groups all showed a significant difference (P<0.05), with the lowest Pdi in the 100% PCV group. As the PCV intensity increased, the Pdi max gradually decreased, showing the same tendency as the Pdi (Fig 2B). As the PCV intensity increased, the Pdi/Pdi max also increased: Particularly when the intensity reached 40% and above, significant changes were observed (Fig 2C).

**Fig 3:**
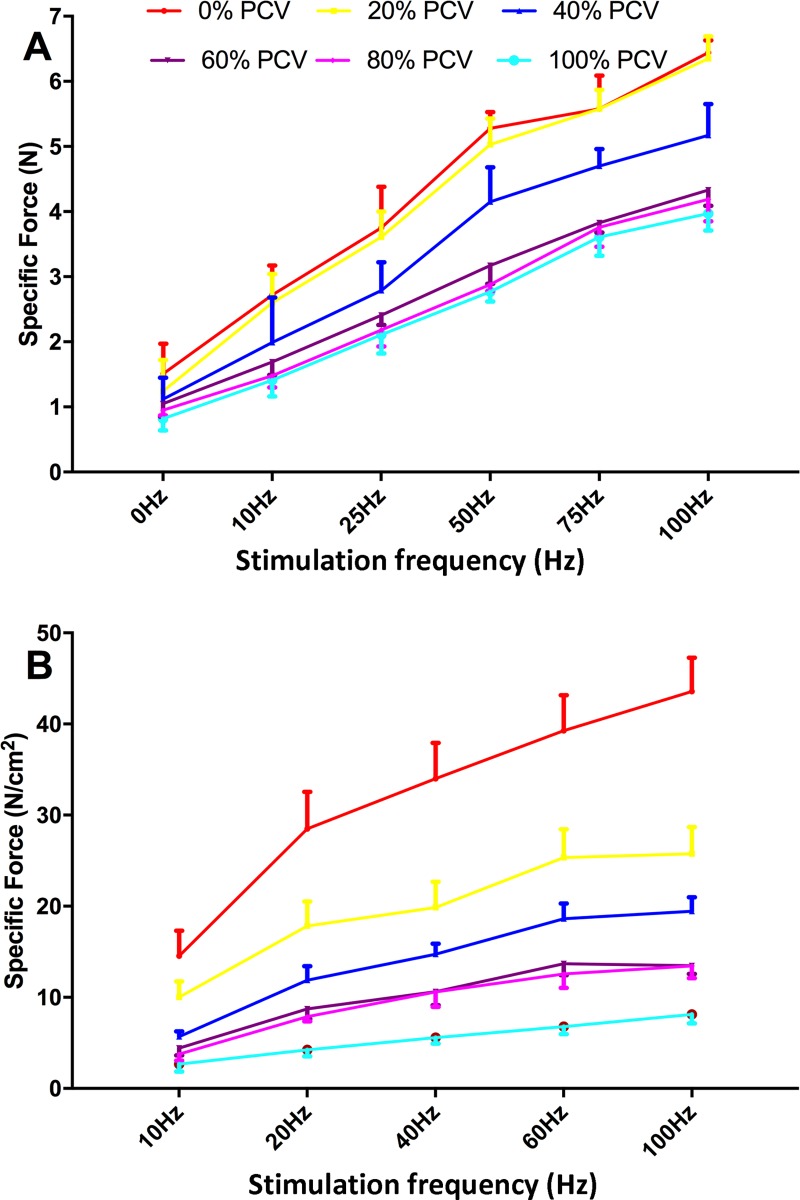
*In vivo* (A) and *in vitro* (B) muscle tonus after 24-h controlled mechanical ventilation at different intensities under different frequencies of electric stimulation.

Under any frequency of electric stimulation, the in vivo muscle tonus was noticeably weakened with the increase in the PCV intensity (Fig 3A). From 40% PCV, the in vivo muscle tonus significantly decreased (P<0.05), whereas no significant difference was observed between the 80% PCV group and the 100% PCV group (P>0.05).

Under any frequency of electric stimulation, the in vitro muscle tonus was noticeably weakened with the increase in the CMV intensity (Fig 3B). Under most frequencies of electric stimulation, different PCV groups showed significant differences compared with each other (P<0.05), except for the difference between the 60% and 80% PCV groups (P>0.05).

### Calpain and casepase-3 levels

**Fig 4:**
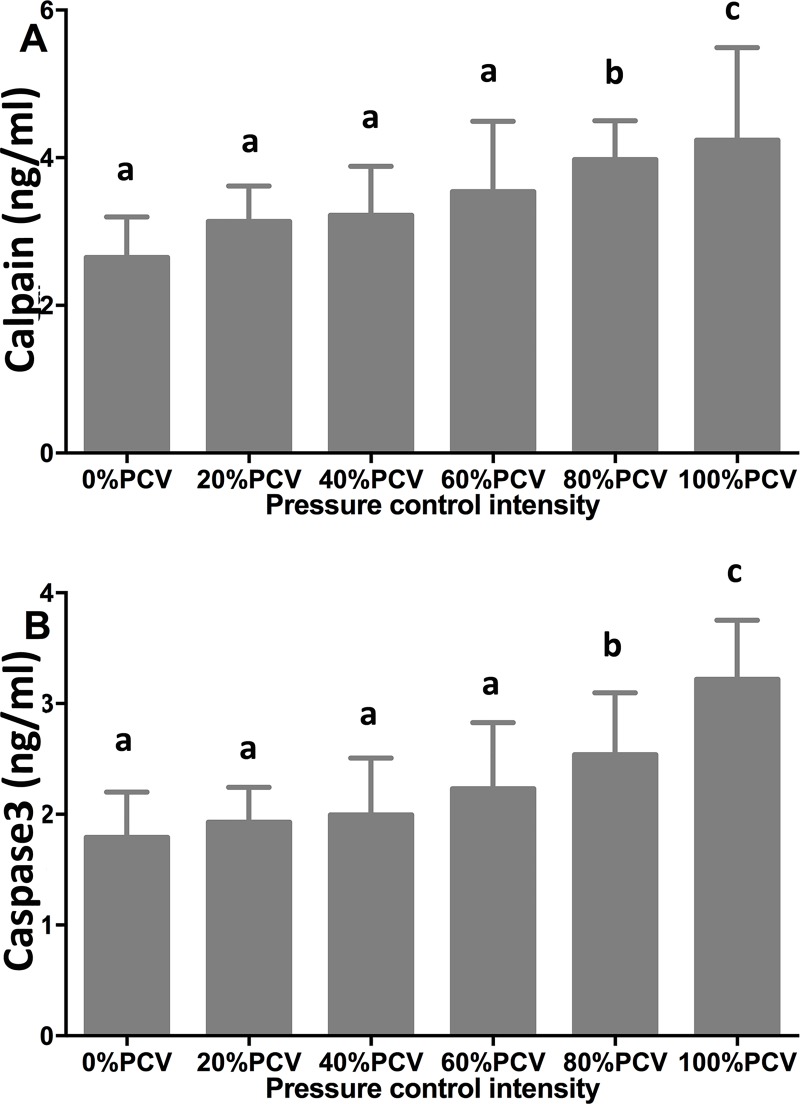
Levels of diaphragmatic calpain (A) and caspase3 (B) after 24-h controlled mechanical ventilation at different intensities.

With the increase in the PCV intensity, the calpain level gradually increased (Fig 4A). Compared with the 0% PCV group, the 80% PCV and above groups showed significant differences (P<0.05), with the highest level observed in the 100% PCV group.

With the increase in the PCV intensity, the casepase-3 level gradually increased (Fig 4B). Compared with the 0% PCV group, the 80% PCV and above groups exhibited significant differences (P<0.05), with the highest level observed in the 100% PCV group.

### The CSAs of the diaphragmatic fibers

**Fig 5:**
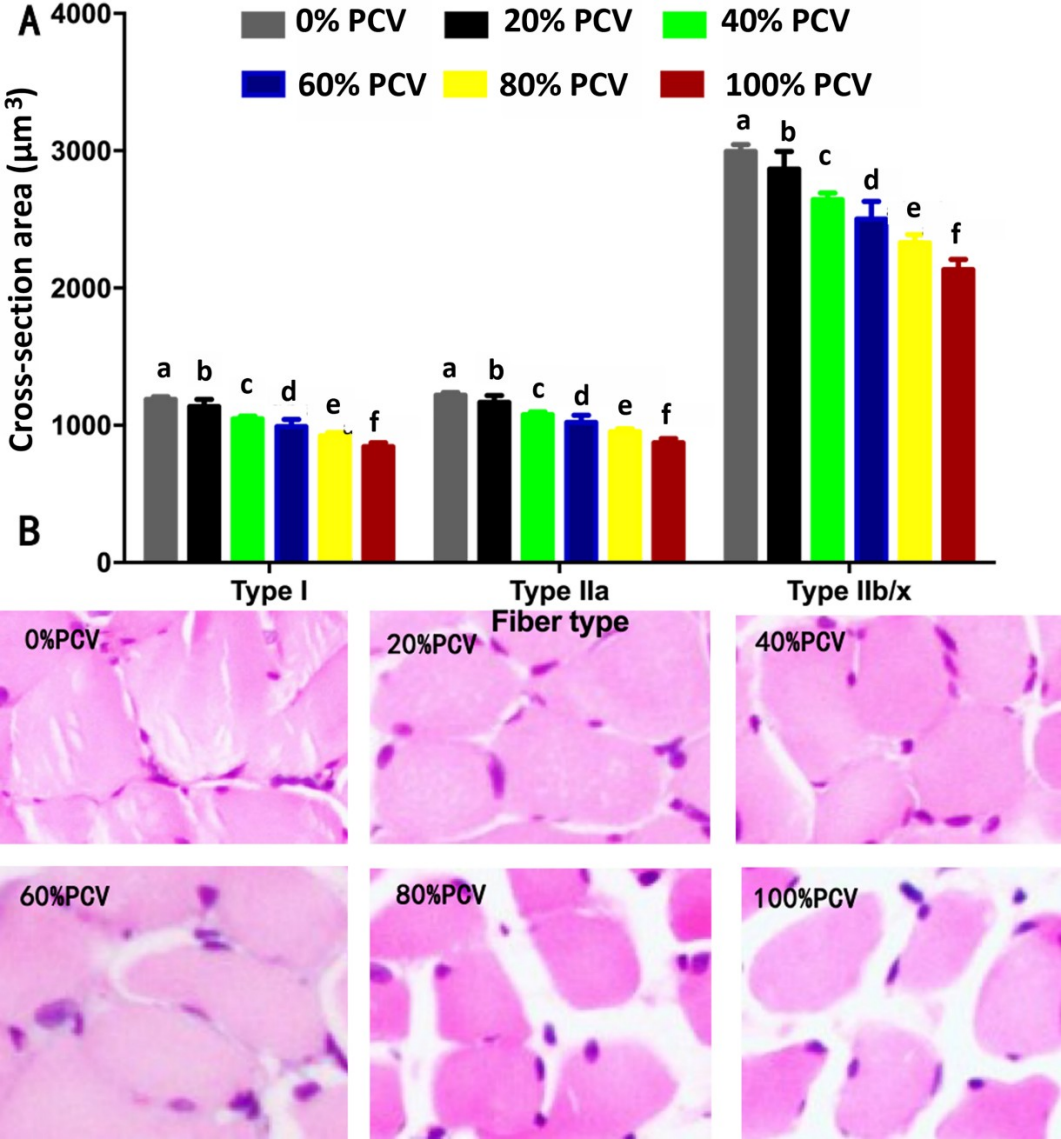
Cross-section areas of diaphragmatic fibers after 24-h controlled mechanical ventilation at different intensities. A, bar chart. B, representative photos under the light microscope (×20). A different letter indicates a significant difference (P<0.05).

With the increase in the PCV intensity, the CSAs of types I, IIa, and IIb/x muscle fibers were all decreased progressively (Fig 5). Pairwise comparisons showed significant differences among all groups, with the least CSAs of all types of muscle fibers observed in the 100% PCV group (all P<0.05).

## Discussion

This study investigated the relationship between SB intensity and VIDD and determined what intensity of SB can effectively prevent the occurrence of VIDD for the first time.

CSAs can visually reflect the variations in the fiber structure of the diaphragm, which has an indicative significance for understanding diaphragmatic function in a dynamic manner. The CSAs of types I, IIa, and IIx/b diaphragmatic fibers all significantly decreased in swine after 72-h CMV; however, such differences were not observed after 72-h adaptive support ventilation [31]. Such a difference might be due to the fact that the activity of the diaphragm can be maintained to some degree during adaptive support ventilation. Another study showed that 6-h CMV did not induce significant atrophy of the diaphragmatic fibers compared with 6-h SB [27]. In this study, the CSAs of the diaphragmatic fibers significantly decreased after 24-h PCV. This finding indicates that CMV can induce early occurrence of diaphragmatic atrophy and suggests that for patients receiving CMV, a ventilation duration less than 24 h may be enough to induce diaphragmatic atrophy. According to a study among patients with brain death, 18–69 hours of full support mechanical ventilation resulted in significant diaphragmatic atrophy [14]. The finding of our study was basically consistent with that reported. Compared to SB, 12-h pressure support ventilation (PSV) caused a significant decrease in the CSA of type IIx/b diaphragmatic fibers in rats; in contrast, 12-h CMV, 18-h CMV, and 18-h PSV all led to significant atrophy of types I, IIa, and IIx/b diaphragmatic muscle fibers [25]. In the PSV mode, as the ventilator is started by the active movements of the diaphragm, a portion of diaphragmatic activity can be maintained. Although PSV can also induce diaphragmatic atrophy, the atrophy-inducing rate was slower than that of CMV. Our study revealed similar outcomes: As PCV strengthened and SB weakened, diaphragmatic activity weakened gradually and diaphragmatic atrophy became worse. These findings indicated that SB intensity was negatively associated with the severity of diaphragmatic atrophy.

Pdi and diaphragmatic tonus reflect diaphragmatic contractility directly and indirectly, respectively. Compared with SB, 24- and 72-h CMV decreased the Pdi max dramatically in rabbits (41% and 63%, respectively) [9], which suggests that diaphragmatic contractile dysfunction induced by CMV can occur as early as 24 hours; furthermore, with the prolongation of CMV, the dysfunction aggravates gradually. Our study obtained a similar result: After 24-h 100% PCV, diaphragmatic tonus greatly decreased. Compared with patients who underwent cardiac surgery, patients with brain death exhibited a significant decrease in the diaphragmatic contractility after 48-h complete CMV [32]. In rabbits, 72-h assisted ventilation and 72-h CMV decreased the diaphragmatic tonus by 20% and 41%, respectively [33]. These findings suggest that the severity of diaphragmatic contractile dysfunction induced by assisted ventilation is lower than that by complete CMV, as part of the autonomic activities of the diaphragm can be maintained in the former mode. Our study demonstrated that maintenance of SB at an intensity above 60%-80% did not cause significant diaphragmatic contractile dysfunction compared with complete SB. Furthermore, the results of this study suggest that the less work the diaphragm does, the more severe the induced VIDD may become.

In this study, 24-h 20% PCV induced the increase in caplain and caspase-3, which was consistent with that reported in literature [34]. Caplain and caspase-3 promote each other [35, 36], that is, the inhibition of one of them will cause a decrease in the level of the other [26]. However, SegoleneMrozek et al reported that the activity of caspase-3 noticeably increased after 6-h CMV, whereas the calpain level did not show a significant change [37], which suggests that the variation in the activity of caspase-3 may occur earlier than that of calpain [37]. What is worth noticing in this study is that although the levels of caplain and caspase-3 increase synchronously with the increase in the proportion of PCV, significant differences were observed until 80% PCV was reached, compared with the 0% PCV; however, significant differences in the diaphragmatic tonus and the CSAs of the diaphragmatic fibers were observed at 40% and 20% PCV, respectively. Presumably, these inconsistencies were caused by the following reasons. First, the measurement equipments employed in this study were not sufficiently sensitive to timely detect the variations in the activity of these proteases. Second, also more important, the decrease in protein synthesis, besides the acceleration of protein degradation, plays an important role in the development of diaphragmatic dysfunction: CMV for 6–18 h significantly decreases the synthesis of diaphragmatic proteins [38, 39]. In the 40% and 60% PCV groups, both protein degradation and protein synthesis inhibition might contribute to the decrease in diaphragmatic tonus, whereas in the 80% and 100% PCV groups, protein degradation alone was sufficient to induce diaphragmatic contractility dysfunction.

This study had the following limitations. First, it employed rat models, and therefore, the results of this study need to be validated by clinical trials. Second, rats have a relatively small body compared to large animals, for which the errors of the results in this study might be large. Third, all animals used in this study were healthy. However, in clinical practice, most patients may suffer from severe diseases, such as sepsis and acute respiratory distress syndrome, which consequently affect the diaphragmatic function. In such conditions, the obtained appropriate PCV intensity in this preliminary study is likely to vary according to the balance between loading and unloading in the patients. Therefore, further experiments to address this limitation will be of great value. Fourth, for patients complicated with chronic respiratory disorder, such as chronic obstructive pulmonary disease, the complications themselves can lead to diaphragmatic atrophy and weakness, which may make VIDD simulation more difficult to realize.

To draw a conclusion, PCV at an intensity of 20% and above can induce diaphragmatic atrophy, an intensity of 40% and above can lead to diaphragmatic contractile dysfunction, and an intensity of 80% and above can result in significant increase in the levels of diaphragmatic proteases. SB intensity is negatively associated with VIDD, and maintenance of SB at an intensity of 60%-80% may have a satisfactory preventative effect on VIDD.
